# Effects of moisture content and tillage methods on creep properties of paddy soil

**DOI:** 10.1371/journal.pone.0253623

**Published:** 2021-06-24

**Authors:** Guoyang Liu, Junfang Xia, Kan Zheng, Jian Cheng, Jun Du, Dong Li

**Affiliations:** College of Engineering, Huazhong Agricultural University, Wuhan, China; Beijing University of Technology, CHINA

## Abstract

The rheological properties parameters of paddy soil affect the interaction between the tillage tools and soil, thus influencing the operation quality and power consumption. In order to study the effects of tillage methods and moisture content on the rheological properties parameters of paddy soil in the middle and lower reaches of the Yangtze River, uniaxial compression creep tests of paddy soils with four moisture contents under no tillage (moisture contents: 26.71%, 24.52%, 23.26%, 21.28%) and plough tillage (moisture contents: 26.77%, 25.55%, 23.40%, 20.56%) were carried out using a TMS-PRO texture analyzer. The creep properties curves obtained from the tests, and the rheological constitutive equation of paddy soil under compression was established by Burgers viscoelastic model. Respectively, the quantitative change rules of creep properties of paddy soil with different moisture contents under different tillage methods and the correlation between these parameters were explored. The results showed that the moisture content under the three-year plough tillage and no tillage methods had significant influence on the rheological properties parameters of paddy soil (*P* < 0.05). The instantaneous elastic modulus, delay elastic modulus, and viscosity coefficient of the two paddy soils (no tillage and plough tillage soils) decreased with the increase of moisture content. However, the variation rules of relaxation time and delay viscosity coefficient with moisture content differed between these two paddy soils. Specifically, the strain rate of the two paddy soils decreased as moisture content decreased, where the total strain combines elastic strain, viscous strain, and viscoelastic strain. The initial strain rate and steady strain rate of the plough tillage paddy soils were lower than that of the no tillage paddy soils. The established creep model equation could be used to obtain viscoelastic rheological parameters of paddy soil in a wide range. The fitting equations between rheological parameters and moisture content were introduced into Burgers model, and the coupling equations between creep deformation and moisture content and time were derived, which could be used to predict the creep properties and deformation behavior of paddy soil in a certain range of no tillage and ploughed field. Overall, this study has a certain theoretical significance for the development and improvement of paddy soil rheology theory, and can also provide theoretical basis and technical support for the research of agricultural machinery design optimization, field water, soil conservation, soil tillage and compaction related simulation analysis in the middle and lower reaches of the Yangtze River.

## Introduction

Soil is a three-phase porous mixture composed of solid particles, liquid water, and air [1–3]. Studying the interaction between soil and tillage tools significantly affects the tillage resistance and work quality of agricultural machinery and is, thus, evaluated in designing and optimizing tillage machines [4–6]. However, the tillage process is very complicated due to the complexity of soil physical composition [7], dynamic factors of tillage machinery [8], and adhesion and fragmentation of soil particles [9]. The creep properties of soil, as a complex rheological body [10], are mainly affected by tillage methods, soil composition, moisture content, environmental temperature, among other factors. Soil rheology, the study of material flow, is essential in the research and development of tillage equipment and formulation of agronomy. The results of such can introduce new ideas for optimizing the design tillage machinery with the aim to reduce drag and consumption and eliminate adhesion and desorption.

At present, studies on the creep properties of agricultural materials mainly focus on fluidity, rheological models, and plant materials, with scarce attention on the rheological properties parameters in farmland soil. For example, Tabatabaeefar [11] reported the accumulation angle of wheat grains of different varieties with a moisture content of 0~22%, the friction coefficient between wheat grains and plywood, glass, plastic, stainless steel, and other materials, and the varied friction properties of wheat grains of different varieties and moisture content. Engelund [12] studied the influence of moisture content and temperature on the tensile creep and deformation recovery of Norway spruce, discovering that both temperature and moisture content affected the tensile creep and recovery by influencing the formation and fracture of hydrogen bonds. Hassani et al. [13] established a 3D anisotropic viscoelastic-plastic rheological model of wood and a secondary development based on the finite element method (FEM), which verified the reliability and accuracy of the model through experiments. In order to predict the cumulative permanent deformation of saturated subgrade soils in Southern China, Zhang et al. [14] explored the effects of moisture content, number of cyclic loading, deviatoric stress and confining pressure on the cumulative permanent strain evolution of saturated subgrade soil in Southern China through long-term cyclic triaxial tests. Based on this, the prediction model of saturated subgrade soil permanent deformation including number of cyclic loading, initial bulk stress, octahedral shear stress and saturation was established, and the verification test was carried out.

Creep model, as an important part of rheological theory, is widely used in agricultural engineering. It mainly includes mechanical damage of fruit, creep model construction of agricultural products, mechanical properties of seeds and rheological properties of flour. Ma et al. [15] carried out tensile creep and stress relaxation tests on rice seedling stems, and found that both creep and stress relaxation were coupling processes between elastic dynamics and viscous resistance within rice stalks. Ahmadi et al. [16] used the finite element method to study the dynamic impact to apple, and established the nonlinear viscoelastic model of the apple. Through the simulation, the change rules of deformation with time in the process of apple contacting with a flat rigid plate under different grasping speeds was obtained. Based on the four-element viscoelastic model parameters of rheological objects, Higashimori et al. [17] analyzed the relationship between the applied grasping force and deformation, and proposed a two-phase control strategy to control the resultant deformation, which can reduced the clamping time under the condition of avoiding excessive deformation of objects. By compression creep tests, Qiu et al. [18] obtained creep properties parameters of different varieties and different moisture content of millet grain group, which could provide theoretical basis for low-loss mechanical harvesting, processing and storage and parameter optimization of millet. Ma et al. [19] studied the effect of wheat bran dietary fiber on the rheological properties of wheat dough. The results showed that adding wheat bran dietary fiber could enhance the mechanical strength of dough, but reduced its viscoelastic strain limit.

Altering tillage methods and moisture content significantly affects the creep properties of soil, thus affecting the interaction mode and stress state between tillage tools and soil [20–23]. At present, research on the influence of tillage methods on farmland soil mostly focuses on the physical properties of soil topsoil, such as the dynamic effect of farming systems on soil carbon and nitrogen in southern Brazil [24]. For instance, Yin et al. [25] suggested that no tillage ridge cultivation could thicken the tillage layer, reduce soil disturbance, promote the formation of large aggregates, and improve soil structure and physical-chemical properties. Research on the influence of moisture content on soil mainly examines agricultural water, soil engineering and soil-machine dynamics, including studies on field soil compaction by machines [26, 27] and on field water, soil conservation, and soil strength [28, 29]. With the development of numerical simulation methods and techniques, the discrete element method (DEM) has been used to solve discontinuous media problems in the field of agricultural engineering [30, 31]. For example, the plastic deformation of soil particles with and without cohesive force was studied by the DEM method and provided a reference for the selection of the soil contact model with little cohesive force [32]. Coetzee [33] used the angle of response as the response index and calibrated the friction coefficient between rock particles using Hertz-Mindlin (no slip) model. Numerous researches have investigated the DEM parameter calibration of inviscid materials based on the Hertz-Mindlin (no slip) model [34] and plastic deformed materials using the linear Hysteretic Spring model [35]. However, until Thakur et al. [36] introduced the Edinburgh Elasto-Plastic Adhesion (EEPA) contact model for the first time, there were limited reports on viscoelastic materials. Based on the Hertz contact theory, this model includes granular plasticity and viscosity, which is suitable for simulating farmland soil with heavy viscosity and strong plasticity [37].

Based on the above research progress, there remain few studies on the rheological properties parameters of farmland soil with creep model, especially the lack of systematic and reliable theoretical calculation models for soil creep properties. Understanding the soil creep properties is largely significant to guide the research and development of agronomy and tillage machinery. In the early stage of our research, we studied the physical composition, physical-chemical properties, and plastic-liquid limit index of paddy soil of the paddy-upland rotation area in the middle and lower of the Yangtze River [38, 39]. In this paper, we discuss on the following objectives: (i) getting the rheological properties parameters of paddy soil by creep tests, (ii) to further analyze the deformation rate, deformation composition, and the correlation of rheological properties parameters, (iii) to forecast the creep properties and deformation behavior of paddy soil in a wide range by the creep model equation of Burgers model, (iv) to deduce the coupling equation between creep deformation and moisture content, time.

## Materials and methods

### Experimental site

**Fig 1:**
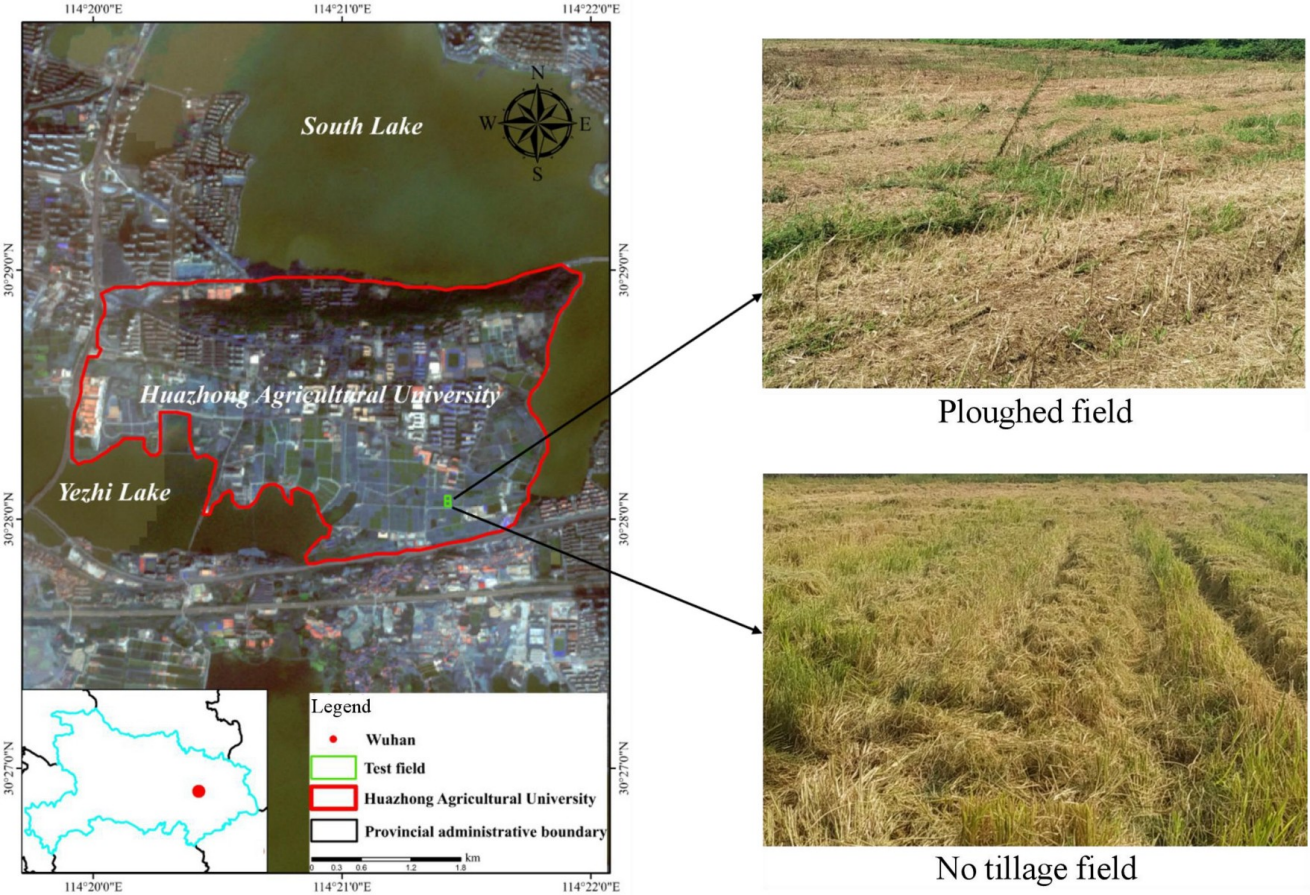
Soil sampling site.

The experiment was conducted from October 2017 to September 2020 in the modern Agricultural Science and Technology Experimental Base of Huazhong Agricultural University (30°28′N, 114°21′E). The base located in Hongshan District, Wuhan City, Hubei province, is characterized by a subtropical moist monsoon climate with seasonal changes in temperature, annual average temperature of 15.8–17.5°C, annual average rainfall of 1000–1500 mm that more concentrated in 6–8 months, annual frost-free period of 211–272 days, and common annual sunshine of 1810–2100 h (Fig 1). The sampling test field is a perennial the paddy-upland rotation area (rice and rape, rice and wheat), containing yellow brown paddy soil and a flat terrain with convenient irrigation and drainage.

### Experimental design

The adopted test was a split plot design, and the employed tillage method was divided into three years of no tillage (NT) and three-year plough tillage (PT). The secondary plot was for the moisture content. Considering the range of moisture content in the cultivation period and the convenience of soil sample preparation, the days of drying field was set 12 days. By varying the days of drying field to changes in the moisture content, each main area was characterized by 4 different moisture contents to make a total of 8 treatments. The four treatments of no tillage field were defined as NTT, NTS, NTN and NTV, corresponding to 3, 6, 9 and 12 days of drying field. Respectively, four treatments of ploughed field were also defined according to the above method, namely PTT, PTS, PTN and PTV.

The effects of moisture content and tillage on the creep properties of paddy soil were studied in two adjacent test plots with an area of 800 m^2^ each. The field had been ploughed for 3 consecutive years with no tillage. On this basis, the zone grouping test was carried out, with each zone covering 20 m^2^ and the groups within the field randomly arranged. The experiment was set to be repeated 5 times. No tillage paddy field was cultivated in semi-drought mode. The field was ditched and ridged according to the direction of water flow, with a ridge width of 80 cm, ditch width of 30 cm, and depth of 30 cm. Water was irrigated in the trench, and the rice was planted on the water level or surface on both sides of the ridge top, with 6 rows planted in each ridge. A conventional transplanter was used for transplanting rice paddy fields in the spring, and topdressing was applied in the earing stage of rice. After the rice harvesting, the soil was ploughed with a share plow (20–25 cm). The straw was turned over and buried in the soil. Then, the surface was exposed, passed through the leisure period, and rotated 3 times before sowing.

### Soil sampling and measurement

#### Sample preparation

**Fig 2:**
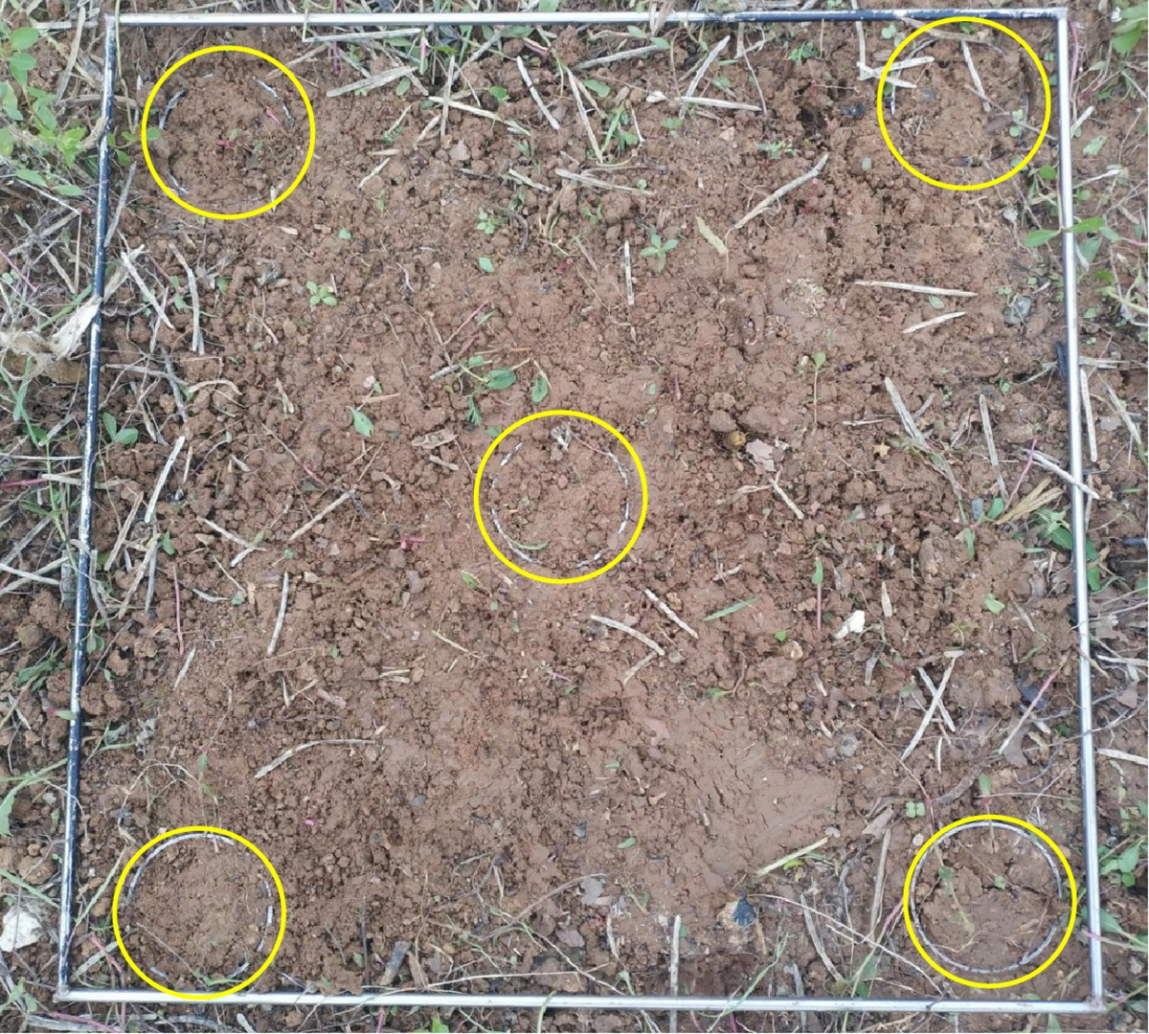
Five point sampling diagram.

A five-point sampling method was used to collect soil samples in the middle rice harvest field of the experimental area (Fig 2). During the experiment, the rice stubble and weeds were removed from the surface of the sampling area. The cultivation layer of 0–10 cm soil in the NT and PT fields was collected by using a ring knife with an inner diameter of 70 mm and height of 52 mm. First of all, we selected the area with flat terrain and uniform straw distribution in the field, placed 1×1 m quadrat in the field to determine the midpoint of the diagonal as the central sampling point, and then select four points on the diagonal with the same distance from the central sampling point as the sampling point. During the soil sampling test, the soil sampling depth of each sampling point was consistent. We sampled many times to measure the mass, removed the samples with large deviation from the test data, and then resampled for measured. Before sampling, a thin layer of Vaseline was applied to the inner wall of the ring knife. The ring knife holder was placed on the ring knife with known weight. We put the edge of the ring knife downward, and then pressed it vertically into the field until the ring knife was full of soil. When the ring knife was pressed in, the force was stable. The scraper was used to cut the soil around the ring knife, and then the ring knife filled with soil was taken out. Finally, the excess soil on both ends and outer wall of the ring knife was removed. Both ends of the ring knife should be covered immediately to avoid water evaporation. Then we measured the weight of samples (accurate to 0.01 g) and recorded it. Basic physical properties of the various soil samples collected from all horizons were analyzed using the standard procedures in Table 1.

**Table 1:** Basic soil physical properties.

Type	Moisture content/%	Organic matter content/(g∙kg^-1^)	Bulk density/(g∙cm^-3^)	Particle composition/%		
				Clay	Powder	Sand
				<0.002mm	0.05~2.0mm	0.05~2.0mm
NTT	26.71	26.78	1.58	41.85	50.63	7.52
NTS	24.52					
NTN	23.26					
NTV	21.28					
PTT	26.77	20.31	1.50	48.56	43.76	7.68
PTS	25.55					
PTN	23.40					
PTV	20.56					

#### Experimental instruments

The TMS-PRO texture analyzer (American FTC company) was used as the main test equipment, which provided a maximum test force of 1000 N and accuracy of ±1%. Other auxiliary tools include the TJSD-750 soil penetrometer/compaction meter (10000 kPa maximum load, ±1% accuracy, 0.1 kPa resolution), Vernier caliper, ring-knife, tape measure, and XMA-600 electric blast drying box (3 kW power, 10–250°C working temperature).

#### Experimental methods

**Fig 3:**
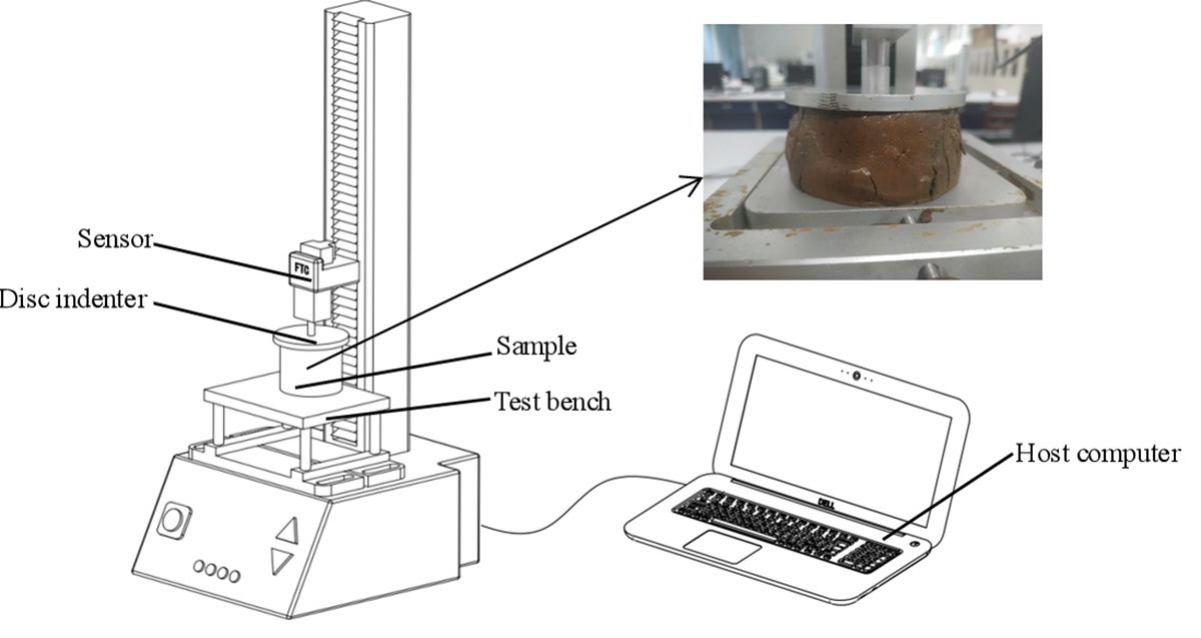
Creep test bench.

Before the test, a pre-experiment was conducted to explore the loads of NT and PT paddy soils under different moisture contents without yield and disintegration. During the test, the sample was fixed onto the test bench. The disk head moved downward at a movement rate of 20 mm/min, and the predetermined load was 60 N, which reached the predetermined load and retained pressure of 900 s. After 900 s, the disk head returned to the initial position automatically, and the strain and time relation were measured. The indoor temperature and humidity were determined to be 25.4 ± 1.5°C and 65.3 ± 4.8%, respectively (Fig 3).

### Statistical analysis

Analysis of variance (ANOVA) and correlation analysis were performed using the SPSS statistical package (Version 21.0 for Windows, SPSS, Chicago, USA) to test the effects of soil moisture content on creep properties. Descriptive statistics, including the mean, standard deviation (SD) and coefficient of variation (CV), were analyzed. Coefficients of the determination (*R*^2^) and root mean squared error (RMSE) were calculated by residual analysis, and WLS (Weighted Least Squares) was adopted to evaluate the performance of different models in fitting the creep properties with moisture content. One-way ANOVA was used with moisture content as the only factor. Differences between the treatments were assessed using their variance and a least significant difference (LSD) multiple comparison test at an *α* = 0.05 level of significance to determine if significant differences existed among the rheological parameters in different moisture content.

## Results

### Determination of creep model

**Fig 4:**
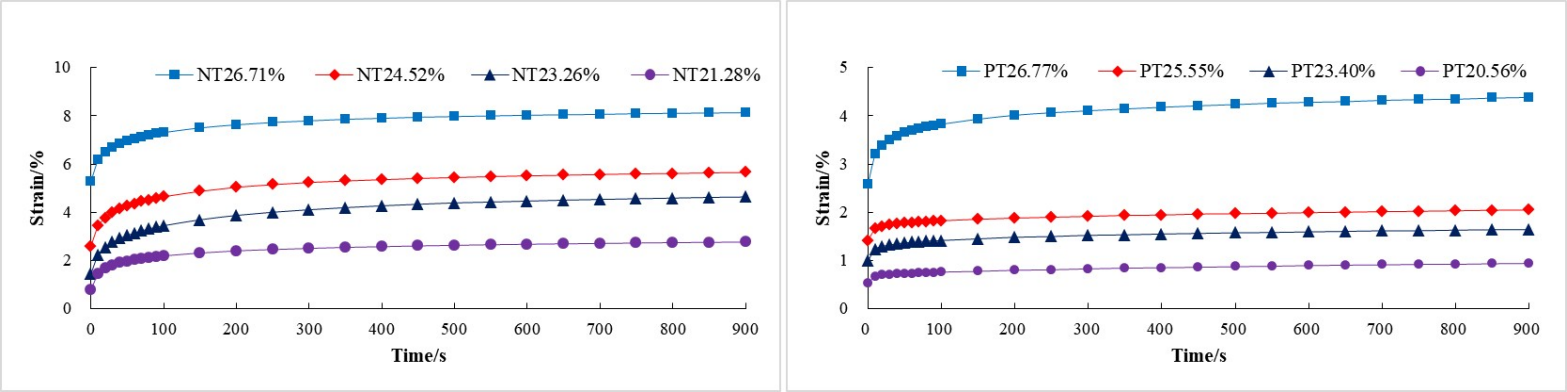
Creep properties curve of paddy soil under different moisture content. (a) No tillage; (b) Plough tillage.

**Fig 5:**
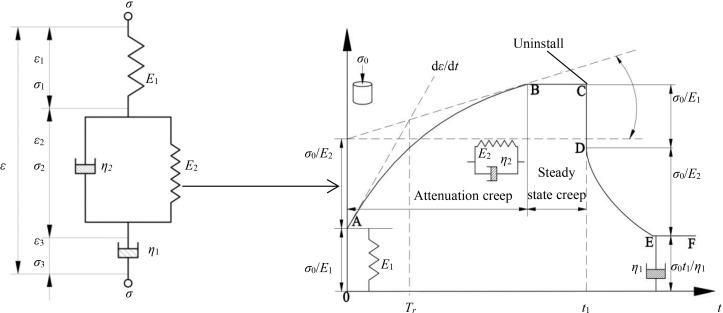
Burgers model creep curve. *σ* is the stress on the Burgers model, *σ = σ*_1_ *= σ*_2_ *= σ*_3_.

The creep curves of NT and PT paddy soils at different moisture content levels were shown (Fig 4). Under the same load, the creep curves of paddy soil of different moisture contents under the two tillage methods were similar. When the applied load was less than the yield strength of the soil, the paddy soil produced an instantaneous strain during the loading process. Since the loading time was shorter than the later compression retaining creep time, the elastic deformation could be considered instantaneous, and its constitutive relationship could be described by elastic. After the elastic deformation, the strain of paddy soil initially increased linearly with time, and then the strain growth rate gradually decreased and eventually approached 0. The strain curve at this stage displayed typical nonlinear properties, and it was difficult to accurately describe its creep properties by using traditional models, such as the Kelvin and Maxwell models [40]. For soils that exhibit typical nonlinear viscoelastic solid-liquid coupling, the existing nonlinear viscoelastic theory does not accurately describe the creep properties, so the linear viscoelastic theory is often needed for precise exploration and analysis [41]. According to the creep curve properties of samples, Burgers model was proposed to study the creep properties of paddy soil, which belonged to linear viscoelastic model, and its mechanical model was shown below (Fig 5).

Burgers model consists of a spring, damper, and the Kelvin model in series (Fig 5). According to the serial properties of the rheological model, under the action of initial stress *σ*_0_, the instantaneous deformation of the spring element *E*_1_ produces strain *σ*_0_/*E*_1_. The damper *η*_1_ fails to produce strain instantly due to the viscosity, while the spring element *E*_2_ fails to produce strain instantaneously due to the viscosity of damper *η*_2_. When the creep stage begins, the Kelvin model and damper *η*_1_ begin to strain under the action of constant stress. After the initial strain is generated, the sample starts to creep at a higher rate, but the strain rate gradually decreases due to the viscous effect of the damper *η*_2_. When the load is discharged at *t*_1_, the spring element *E*_1_ returns to the state of *ε* = 0 instantaneously, while the spring element *E*_2_ in the Kelvin model slowly returns to the initial position due to the viscosity of the damper *η*_2_. However, because the load has been unloaded and the damper *η*_2_ does not return to its original position, the experimental sample permanently deforms.

The relaxation time *T*_r_ = *η*_2_/*E*_2_ is defined, and the constitutive equation of the model is derived according to the stress-strain relationship:

$$\varepsilon =\frac{\sigma }{E_{1}}+\frac{\sigma }{\eta_{1}}t+\frac{\sigma }{E_{2}}(1‐e^{-\frac{t}{T_{r}}})$$ (1)

According to formula (1), when *t* = 0, the elastic element *E*_1_ undergoes instantaneous elastic deformation, *ε* = *σ*/*E*_1_. When *t* goes to infinity, *ε* goes to infinity, and strain rate is determined from the derivative of formula (1) as:

$$\frac{d\varepsilon }{dt}=\frac{\sigma }{\eta_{1}}+\frac{\sigma }{\eta_{2}}e^{-\frac{t}{T_{r}}}$$ (2)

According to formula (2), with the passage of time, the strain rate decreases gradually and finally reaches a constant value, namely d*ԑ*/d*t* approaches *σ*/*η*_1_. Based on the above derivation, Burgers model can accurately describe the instantaneous elastic deformation, delayed elastic deformation, viscous flow, attenuation creep, and steady-state creep of paddy soil under compression creep.

### Fitting and correlation analysis of rheological parameters

According to formula (1), MATLAB was used to fit Burgers model under different moisture contents of NT and PT paddy soil, then the rheological parameters were calculated, which were shown in Table 2. The coefficient of rheological fitting of the paddy soil samples above 0.985 and the coefficient of variation below 0.30% indicate that Burgers model could accurately describe the creep properties of rice soil in the paddy-upland rotation area in the middle and lower reaches of the Yangtze River. The superscripts in the Table 2 were the result of multiple comparisons of rheological parameters of paddy soil by least significant difference (LSD). Different lowercase letters in the same column represented significant differences (*P* < 0.05). The data in the Table 2 were represented by mean and standard deviation.

**Table 2:** Fitting parameters of creep model for no tillage paddy soil.

Type	Moisture content	Instant elastic modulus	Viscosity coefficient	Delay elastic modulus	Delay time	Delay viscosity coefficient	*R*^2^
	*w*/%	*E*_1_/MPa	*η*_1_/MPa·s	*E*_2_/MPa	*T*_r_/s	*η*_2_/MPa·s	
NTT	26.71±0.25	(0.189±0.010)^d^	(577.616±49.945)^c^	(0.333±0.043)^b^	(103.288±9.696)^a^	(34.237±1.222)^c^	0.993
NTS	24.52±0.49	(0.624±0.002)^c^	(2149.552±222.766)^b^	(1.095±0.012)^b^	(72.622±0.075)^bc^	(79.557±0.954)^b^	0.991
NTN	23.26±0.65	(0.959±0.005)^b^	(2469.758±193.923)^b^	(2.077±0.028)^a^	(95.355±14.800)^ab^	(197.896±28.117)^a^	0.989
NTV	21.28±0.87	(1.075±0.016)^a^	(3426.444±185.274)^a^	(2.200±0.559)^a^	(58.589±1.674)^c^	(129.393±36.455)^b^	0.988
PTT	26.77±0.39	(0.418±0.028)^c^	(3135.026±591.146)^c^	(2.411±0.360)^c^	(57.092±1.290)^c^	(137.678±17.437)^c^	0.987
PTS	25.55±0.48	(0.455±0.002)^c^	(7612.308±88.090)^b^	(6.104±1.151)^b^	(54.407±0.209)^bc^	(332.092±61.354)^b^	0.987
PTN	23.40±0.59	(0.886±0.003)^b^	(8861.322±5.552)^a^	(6.761±0.003)^b^	(53.778±0.143)^b^	(363.611±1.156)^b^	0.987
PTV	20.56±0.77	(1.642±0.015)^a^	(9414.022±28.200)^a^	(17.164±1.071)^a^	(71.572±1.485)^a^	(1228.459±51.135)^a^	0.993

Note: Different lowercase letters in the same column represent significant differences (*P* < 0.05), and the mean value is used ± Standard error, the same below.

Instantaneous elastic deformation will occur in paddy soil under a load, for which the instantaneous elastic modulus and delay elastic modulus represent the elastic mechanical properties of paddy soil. For NT paddy soil, the instantaneous elastic modulus and delay elastic modulus ranged 0.189–1.075 MPa and 0.333–2.200 MPa with average values of 0.712 and of 1.427 MPa, respectively. For PT paddy soil, the instantaneous elastic modulus and delay elastic modulus ranged 0.418–1.642 MPa and 2.411 to 17.164 MPa with average values of 0.850 MPa and 8.110 MPa, respectively. The viscosity coefficient characterizes the fluidity of paddy soil, where a larger value indicates a stronger anti-deformation viscosity resistance and poor fluidity. Relaxation time, also known as elastic lag time, is the result of the comprehensive action of the delayed elastic modulus *E*_2_ and delayed viscosity coefficient *η*_2_. For NT paddy soil, the viscosity coefficient, delayed viscosity coefficient, and relaxation time ranged 577.616–3426.444 MPa·s, 34.237–129.393 MPa·s, and 58.589–103.288 s with average values of 2155.842 MPa·s, 110.271 MPa·s, and 82.463 s, respectively. In comparison, PT paddy soil exhibited a viscosity coefficient of 3135.026–9414.022 MPa·s (average of 7255.669 MPa·s), relaxation time of 57.092–71.572 s (average of 59.212 s), and delayed viscosity coefficient of 137.678–1228.459 MPa·s (average of 515.460 MPa·s). The above analysis shows that in the internal structure of PT paddy soil and NT paddy soil, the elastic deformation resistance of the spring element *E*_1_ is less than that of spring element *E*_2_ in the Kelvin model. Also, the viscous resistance to deformation of the viscous element *η*_1_ is stronger than the delayed viscous element *η*_2_ in the Kelvin model. Moreover, the fluidity of the internal structure of PT paddy soil is weaker than that of NT soil.

The mean values of rheological mechanical parameters of the two paddy soils were compared within 12 days. The instantaneous elastic modulus, delay elastic modulus, viscosity coefficient, and delayed viscosity coefficient of NT paddy soil were lower than those of PT paddy soil. The relaxation time of NT paddy soil was higher than that of PT paddy soil. From the aspect of moisture content, the instantaneous elastic modulus, viscosity coefficient, and delay elastic modulus of the two paddy soils decreased as moisture content increased, and the maximum was obtained by NTV and PTV treatments. The variation trend of relaxation time and delayed viscosity coefficient of the two paddy soils were different with moisture content. The maximum value of relaxation time was due to the NTT, PTV treatment, while the maximum delayed viscosity coefficient was attributed to the NTV, PTV treatment.

ANOVA was performed for NT and PT paddy soils with different moisture contents (Table 3). The moisture content exhibited a significant influence on the instantaneous elastic modulus, delay elastic modulus, viscosity coefficient, and delayed viscosity coefficient of NT (*P* < 0.01) and had a significant influence on the relaxation time of NT (*P* < 0.05). Moisture content also demonstrated a significant effect on the instantaneous modulus of elasticity, delay elasticity modulus, viscosity coefficient, delayed viscosity coefficient, and relaxation time of PT paddy soil (*P* < 0.01).

**Table 3:** Paddy soil variance analysis.

Source	Type	Sum of square	Degree of freedom	Mean square	*F*	*P*
*E*_1_	NT	0.949	3	0.316	3.395×10^3^	0.00
*η*_1_		8.307×10^6^	3	2.803×10^6^	90.367	0.00
*E*_2_		4.654	3	1.551	19.662	0.01
*T*_r_		2.533×10^3^	3	844.472	10.695	0.02
*η*_2_		2.954×10^4^	3	9.845×10^3^	18.559	0.01
*E*_1_	PT	1.942	3	0.647	2.473×10^3^	0.00
*η*_1_		4.869×10^7^	3	1.623×10^7^	181.310	0.00
*E*_2_		240.581	3	80.194	123.335	0.00
*T*_r_		419.757	3	139.919	142.247	0.00
*η*_2_		1.414×10^6^	3	4.712×10^5^	281.986	0.00

Note: the *P* value in the table is accurate to two decimal places, and 0.00 in the table means that the *p* value is far less than 0.01.

**Fig 6:**
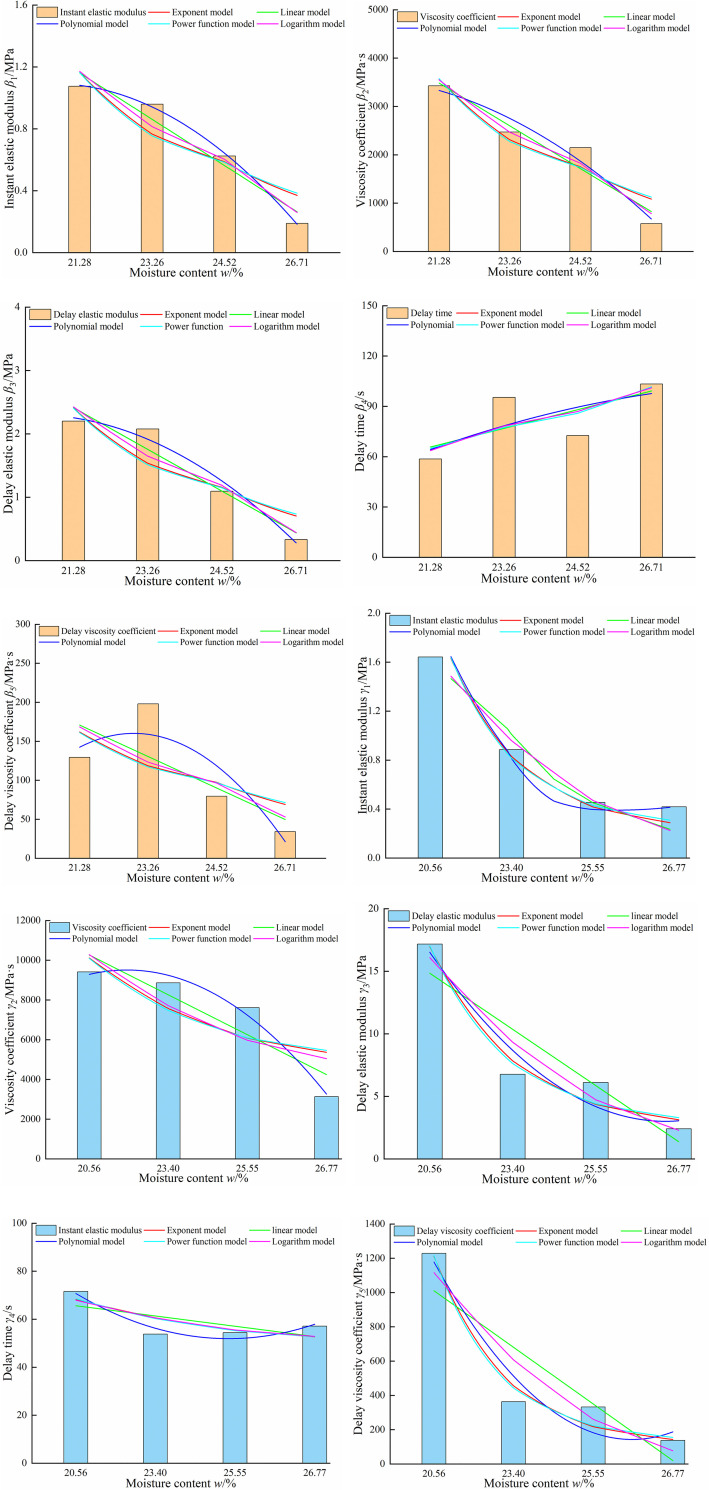
Variation of rheological mechanical parameters under different moisture contents. (a) Instant elastic modulus of no tillage field, (b) Viscosity coefficient of no tillage field, (c) Delay elastic modulus of no tillage field, (d) Delay time of no tillage field, (e) Delay viscosity coefficient of no tillage field, (f) Instant elastic modulus of plough field, (g) Viscosity coefficient of plough field, (h) Delay elastic modulus of plough field, (i) Delay time of plough field, (j) Delay viscosity coefficient of plough field.

To construct the model between moisture content and rheological mechanical parameters, the most basic mathematical and five most widely-used functional relationships were selected, i.e. polynomial, logarithmic, exponential, power function, and linear models. A numerical analysis method and SPSS Statistics 21.0 software were used to explore the quantitative change relationship between the moisture content and rheological parameters of paddy soil. The polynomial, logarithmic model, exponential, power function, and linear relationships between moisture content and rheological parameters of paddy soil were obtained by fitting, as shown in Fig 6. The optimal model fitting results of various rheological parameters and moisture content were shown in Table 4.

**Table 4:** Optimal fitting model of relationship between rheological mechanical parameters and moisture content of paddy soil.

Type	Parameter	Model	Fitting model expression	*F*	*P*	*R*^2^
NT	*β*_1_	Polynomial model	*β*_1_ = -0.08*w*^2^+0.1*w*+1.061	1297.796	0.000	0.998
	*β*_2_	Polynomial model	*β*_2_ = -153.812*w*^2^-117.608*w*+3603.454	41.882	0.001	0.944
	*β*_3_	Polynomial model	*β*_3_ = -0.160*w*^2^+0.140*w*+2.274	26.258	0.002	0.913
	*β*_4_	Power function model	*β*_4_ = 62.082*w*^0.324^	6.633	0.042	0.525
	*β*_5_	Polynomial model	*β*_5_ = -28.456*w*^2^+101.897*w*+68.945	6.418	0.042	0.720
PT	*γ*_1_	Polynomial model	*γ*_1_ = 0.180*w*^2^-1.308*w*+2.774	3186.302	0.000	0.999
	*γ*_2_	Polynomial model	*γ*_2_ = 981.146*w*^2^+2897.128*w*+7371.442	120.221	0.000	0.980
	*γ*_3_	Logarithm model	*γ*_3_ = -10.182ln(*w*)+16.20	73.356	0.000	0.924
	*γ*_4_	Polynomial model	*γ*_4_ = 5.120*w*^2^-29.880*w*+95.514	31.979	0.001	0.927
	*γ*_5_	Power function model	*γ*_5_ = 1194.020*w*^-1.452^	77.239	0.000	0.928

Note: *β*_1_-*β*_5_ corresponding to instantaneous elastic modulus *E*_1_, viscosity coefficient *η*_1_, delayed elastic modulus *E*_2_, delay time *T*r and delayed viscosity coefficient *η*_2_ (no tillage soil); *γ*_1_- *γ*_5_ corresponding to instantaneous elastic modulus *E*_1_, viscosity coefficient *η*_1_, delayed elastic modulus *E*_2_, delay time *T*r and delayed viscosity coefficient *η*_2_ (ploughed soil), the same below.

Table 4 revealed relatively low determining coefficients of the fitting equation for the relaxation time and delay viscosity coefficient of NT paddy soil, which optimally fit the power function model and polynomial model, respectively. Other determining coefficients of the optimal fitting model of rheological parameters of paddy soil all exceeded 0.91, and the corresponding *F* value of the optimal fitting model was greater than the critical value of *F*_0.05_. At the same time, the *P* value of the model was less than 0.05, which indicated that it passed the significance test of the regression model. Comprehensive analysis of the above test results revealed differences between the optimal fitting models of the rheological parameters and moisture content of NT and PT paddy soils.

The large standard deviation of fitting parameters in Table 2 and the difference of fitting models in Table 4 reflected the complexity of soil components. This indicated that in the process of agricultural production, different field conditions and the uneven distribution of water, fertilizer, and other resources would cause variations in the soil structure, leading to a large degree of dispersion among the rheological parameters of paddy soil. Therefore, correlation analysis was performed on the creep model parameters of paddy soil specified in Table 2, and the results were shown in Table 5.

**Table 5:** Correlation of paddy soil creep parameters.

Type	Parameter	*E*_1_*/*Instant elastic modulus	*η*_1_*/*Viscosity coefficient	*E*_2_*/*Delay elastic modulus	*T*_r_*/*Delay time	*η*_2_*/*Delay viscosity coefficient
NT	*E*_1_	1	—	—	—	—
	*η*_1_	0.959**	1	—	—	—
	*E*_2_	0.966**	0.880**	1	—	—
	*T*_r_	-0.598	-0.745*	-0.497	1	—
	*η*_2_	0.826*	0.661	0.883**	-0.081	1
PT	*E*_1_	1	—	—	—	—
	*η*_1_	0.700	1	—	—	—
	*E*_2_	0.864**	0.736*	1	—	—
	*T*_r_	0.954**	0.342	0.871**	1	—
	*η*_2_	0.956**	0.668	0.994**	0.918**	1

Note:

**indicates the extreme significant difference (*P* < 0.01)

*indicates the significant difference (*P* < 0.05).

For NT paddy soil, the instant elastic modulus *E*_1_, viscosity coefficient *η*_1_, and delay elastic modulus *E*_2_ were very significantly positive correlated (*P* < 0.01). The instant elastic modulus *E*_1_ and delay viscosity coefficient *η*_2_ were also significantly positive correlated (*P* < 0.05). For PT paddy soil, the instantaneous elastic modulus *E*_1_ was very significantly positively correlated with significant delay elastic modulus *E*_2_, relaxation time *T*_r_, and delay viscosity coefficient *η*_2_ (*P* < 0.01). The results suggested that when the instantaneous elastic modulus *E*_1_ of paddy soil was larger, the delay elastic modulus *E*_2_ was larger, and the stable creep of paddy soil was faster. In other words, a greater instantaneous elastic modulus *E*_1_ of NT paddy soil means a greater viscosity coefficient *η*_1_ and delay viscosity coefficient *η*_2_, reflecting that viscosity increases with a greater elasticity and worse internal mobility of paddy soil. Moreover, the viscosity coefficient *η*_1_ of NT paddy soil was very significantly positively correlated with the delay elastic modulus *E*_2_ (*P* < 0.01) and negatively correlated with relaxation time *T*_r_ (*P* < 0.05). The results showed that the stronger viscosity of NT paddy soil, the longer time to reach stable creep. For PT paddy soil, a greater instantaneous elastic modulus *E*_1_ correlated with a higher delay elastic modulus *E*_2_, relaxation time *T*_r_, and delay viscosity coefficient *η*_2_. This reflected that with increased elasticity, the elasticity and viscosity of the Kelvin element in Burgers model were enhanced, further increasing the stable creep time. In addition, a very significant positive correlation was found between delayed elastic modulus *E*_2_ and viscosity coefficient *η*_1_, relaxation time *T*_r_, and delayed viscosity coefficient *η*_2_ (*P* < 0.01). The relaxation times *T*_r_ of NT and PT paddy soil were very significantly correlated with the viscosity coefficient (*η*_1_ or *η*_2_). The bigger the viscosity coefficient *η*_1_ of NT paddy soil was, the smaller the relaxation time *T*_r_. The greater the delay viscosity coefficient *η*_2_ of PT paddy soil was, the greater the relaxation time *T*_r_. The above analysis indicated that there were both positive and negative correlations between the creep model parameters of paddy soil, further reflecting the differences between the creep properties of NT and PT paddy soil.

### Parametric analysis of creep compliance and strain rate

When describing the viscoelasticity of a material in Burgers model, the creep flexibility *J*(*t*) is used to represent the creep energy and creep strain of the material under the action of unit stress, which can be expressed as:

$$J(t)=\varepsilon (t)/\sigma_{0}=J_{e}+J_{v}(t)+J_{\mathrm{ve}}(t)$$ (3)

where *J*_e_ stands for elastic compliance; *J*_e_ = 1/*E*_1_; *J*_v_(*t*) represents viscosity compliance; J_v_(*t*) = *t*/*η*_1_; *J*_ve_(*t*) represents the viscoelastic compliance; *J*_ve_(*t*) = [1-exp(-*E*_2_*t*/*η*_2_)]/*E*_2_, *σ*_0_ represents the stress on the material; and *t* is time of applied stress. According to the expression of creep compliance *J*(*t*), the elastic compliance *J*_e_ is the instantaneous elastic deformation under the stress applied to paddy soil, which does not change with time. The coefficient of viscosity compliance *J*_v_(*t*), which increases slowly and linearly with time, is the reciprocal of the coefficient viscosity *η*_1_, where the creep curve shows the creep rate of uniform creep stage of paddy soil. The viscoelastic compliance *J*_ve_(*t*) is influenced by the delay elastic modulus *E*_2_ and delay viscosity coefficient *η*_2_. As time increases, the viscoelastic compliance *J*_ve_(*t*) gradually increases, then the growth rate slows down to a fixed value. The creep curve illustrates the decelerating creep stage of paddy soil.

The creep curve fitting results of NT and PT paddy soil in Table 2 were transformed according to formula (1) to obtain the components of total creep strain of both soils at each moisture content level. The specific expressions are as follows:

$$\left\{ \begin{array}{l} \varepsilon_{1}(t)=0.106+3.463\times 10^{‐5}t+0.060\times (1-e^{-t/103.288})\\ \varepsilon_{2}(t)=0.032+9.304\times 10^{‐6}t+0.018\times (1-e^{-t/72.622})\\ \varepsilon_{3}(t)=0.021+8.098\times 10^{‐6}t+9.629\times 10^{‐3}\times (1-e^{-t/95.355})\\ \varepsilon_{4}(t)=0.019+5.837\times 10^{‐6}t+9.091\times 10^{‐3}\times (1-e^{-t/58.589})\\ \varepsilon_{5}(t)=0.048+6.380\times 10^{‐6}t+8.294\times 10^{‐3}\times (1-e^{-t/57.092})\\ \varepsilon_{6}(t)=0.044+2.627\times 10^{‐6}t+3.277\times 10^{‐3}\times (1-e^{-t/54.407})\\ \varepsilon_{7}(t)=0.023+2.567\times 10^{‐6}t+2.958\times 10^{‐3}\times (1-e^{-t/53.778})\\ \varepsilon_{8}(t)=0.012+2.124\times 10^{‐6}t+1.165\times 10^{‐3}\times (1-e^{-t/71.572}) \end{array}\right.$$ (4)

Where *ε*_1_(*t*), *ε*_2_(*t*), *ε*_3_(*t*), *ε*_4_(*t*), *ε*_5_(*t*), *ε*_6_(*t*), *ε*_7_(*t*), and *ε*_8_(*t*) correspond to NTT, NTS, NTN, NTV, PTT, PTS, PTN, and PTV treatments. By substituting the fitting model between moisture content and rheological mechanical parameters of paddy soil in Table 4 into formula 1, the coupling mathematical model between creep deformation, moisture content and time of no tillage soil and ploughed soil can be obtained.


$$\left\{ \begin{array}{l} \varepsilon_{\mathrm{NT}}(w,t)=\frac{\sigma }{0.08w^{2}+0.1w+1.061}+\frac{\sigma }{‐153.812w^{2}‐117.608w+3603.454}t\\ \;+\frac{\sigma }{‐0.160w^{2}+0.140w+2.274}(1‐e^{‐\frac{t}{62.082w^{0.324}}})\\ \varepsilon_{\mathrm{PT}}(w,t)=\frac{\sigma }{0.180w^{2}‐1.308w+2.774}+\frac{\sigma }{‐10.182\ln(w)+16.20}t\\ \;+\frac{\sigma }{‐981.146w^{2}+2897.128w+7371.442}(1‐e^{‐\frac{t}{5.120w^{2}‐29.880w+95.514}}) \end{array}\right.$$ (5)

It can be seen that the total creep deformation of paddy soil consists of elastic strain *ε*_e_(*t*), viscous strain *ε*_v_(*t*), and viscoelastic strain *ε*_ve_(*t*). The proportion of each strain in the total strain was further analyzed using the following proportion:

$$P_{i}(t)=\frac{\varepsilon_{i}(t)}{\varepsilon (t)}\times 100\%=\frac{J_{i}(t)}{J_{e}(t)+J_{\mathrm{ve}}(t)+J_{v}(t)}\times 100\%$$ (6)

**Fig 7:**
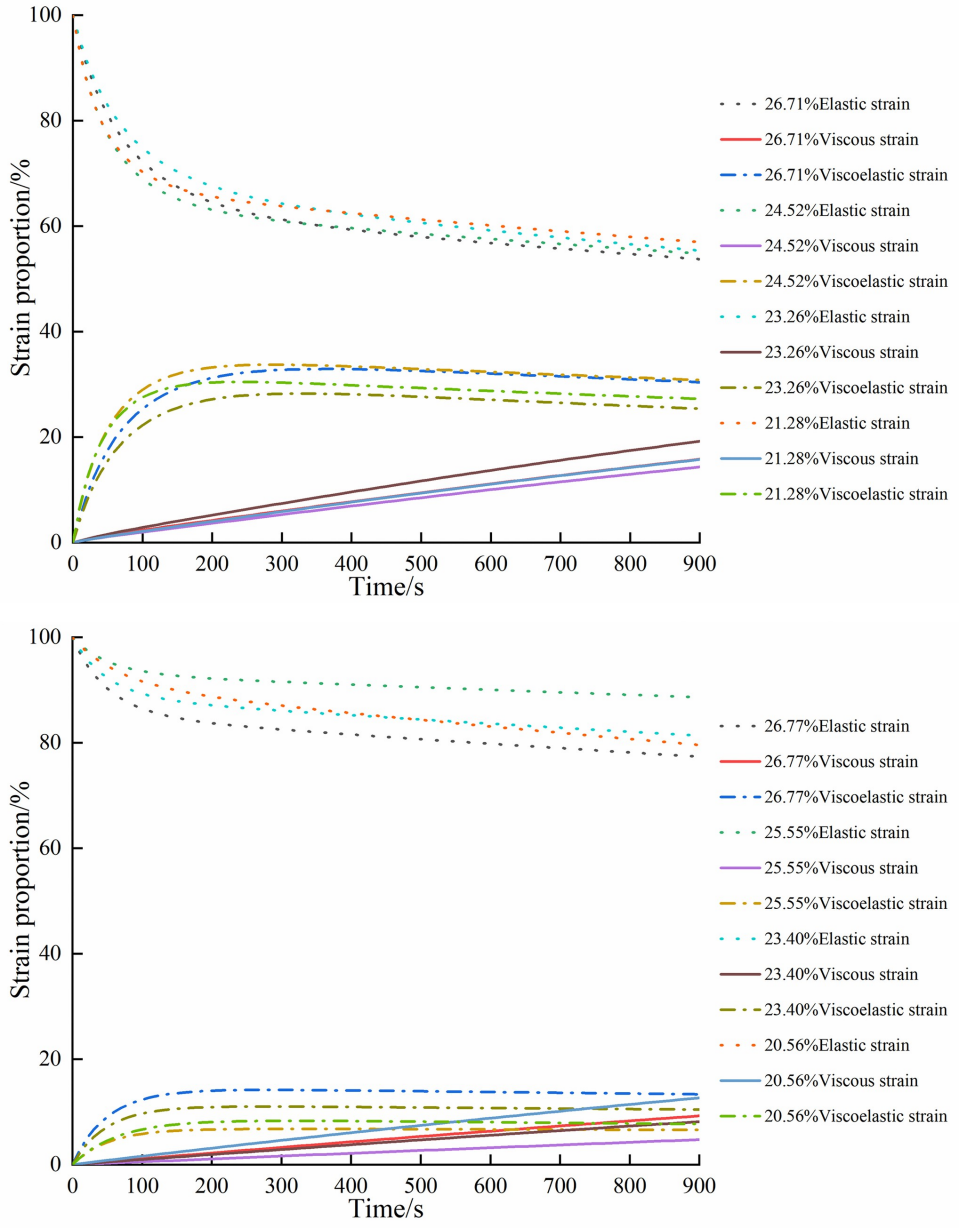
Strain proportion under each moisture content. (a) No tillage; (b) Plough tillage.

Fig 7 displays the change in strain proportion with time under each moisture content of paddy soil. Specifically, the proportion of elastic strain in the total strain exhibited an initially fast nonlinear decrease that gradually slowed with time and consistent decrease with the increase of moisture content. The proportion of viscous strain in the total strain increased as creep time increased with a linear correlation coefficient inversely proportional to moisture content. The proportion of viscoelastic strain in the total strain first increased then decreased with time. When NT paddy soil reached the steady-state creep stage, the elastic strain corresponding to NTV accounted for the largest proportion of the total strain. The viscous strain corresponding to NTN accounted for the largest proportion of the total strain, and the proportion of viscoelastic strain in the total deformation was the smallest. The viscoelastic strain corresponding to NTS contributed the largest proportion of the total strain, while viscous strain composed the smallest proportion. The proportion of elastic strain of NTT had the smallest proportion. When PT paddy soil reached the stage of steady creep, the elastic strain corresponding to PTS accounted for the largest proportion of the total strain, and the viscous strain, viscoelastic strain occupied the smallest proportion of the total deformation. The viscoelastic strain corresponding to PTT had the largest proportion of the total strain, while elastic strain accounted for the smallest proportion. The viscous strain corresponding to PTV occupied the largest proportion.

The strain rate is an index that represents the rate of creep change and strain change per unit time, which can be expressed as follows:

$$v(t)=|\frac{d\varepsilon (t)}{dt}|$$ (7)

Using formula (7), the derivatives of each equation in formula (4) were obtained as the following strain rate expressions of paddy soil at each moisture content level:

$$\left\{ \begin{array}{l} v_{1}(t)=3.463\times 10^{‐5}+5.809\times 10^{‐4}e^{-t/103.288}\\ v_{2}(t)=9.304\times 10^{‐6}+2.479\times 10^{‐4}e^{-t/72.622}\\ v_{3}(t)=8.098\times 10^{‐6}+1.010\times 10^{‐4}e^{-t/95.355}\\ v_{4}(t)=5.837\times 10^{‐6}+1.552\times 10^{‐4}e^{-t/58.589}\\ v_{5}(t)=6.380\times 10^{‐6}+1.453\times 10^{‐4}e^{-t/57.092}\\ v_{6}(t)=2.627\times 10^{‐6}+6.023\times 10^{‐5}e^{-t/54.407}\\ v_{7}(t)=2.567\times 10^{‐6}+5.500\times 10^{‐5}e^{-t/53.778}\\ v_{8}(t)=2.124\times 10^{‐6}+1.628\times 10^{‐5}e^{-t/71.572} \end{array}\right.$$ (8)

**Fig 8:**
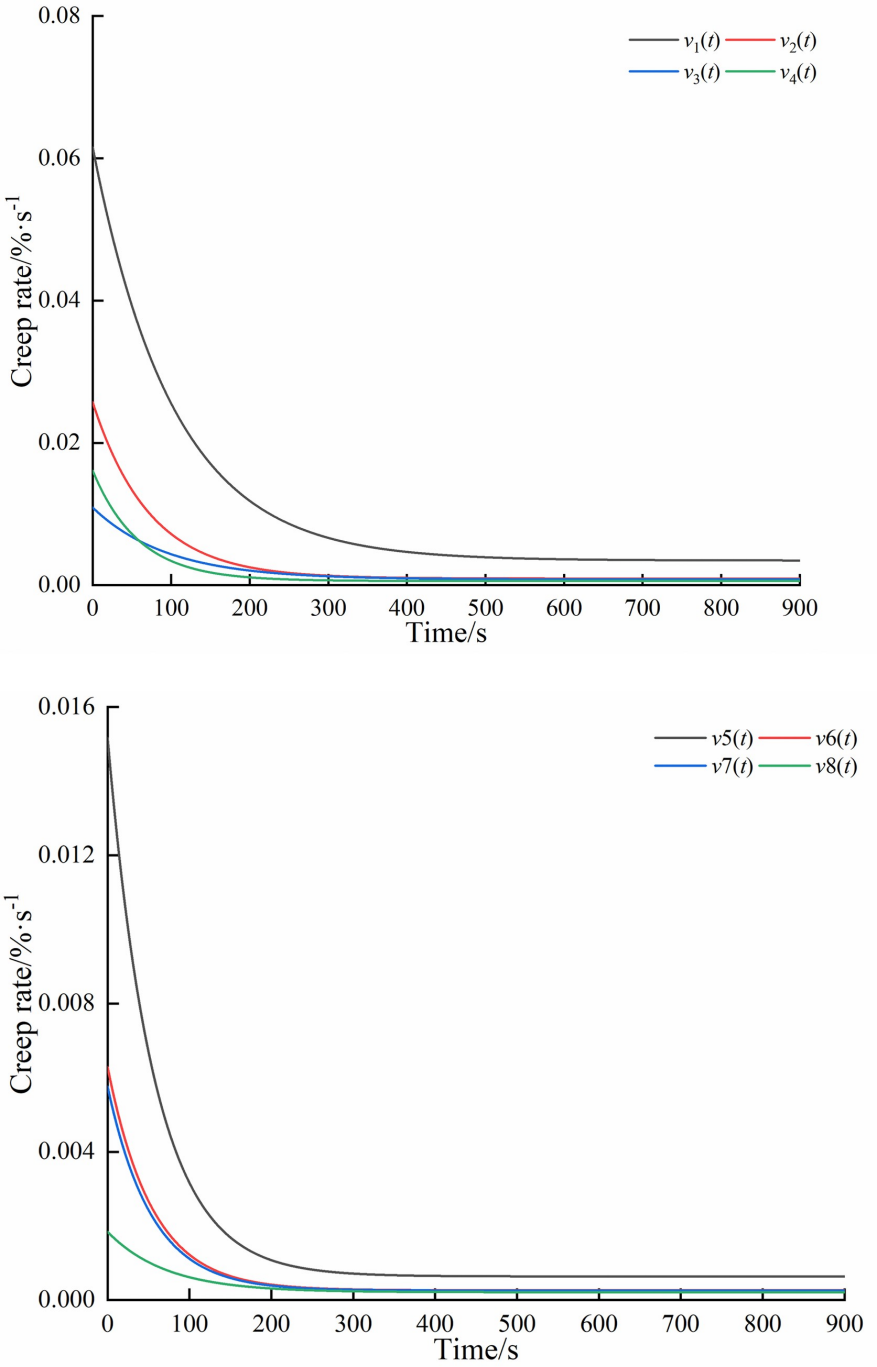
Deformation rate curve under different moisture content. (a) No tillage; (b) Plough tillage.

**Fig 9:**
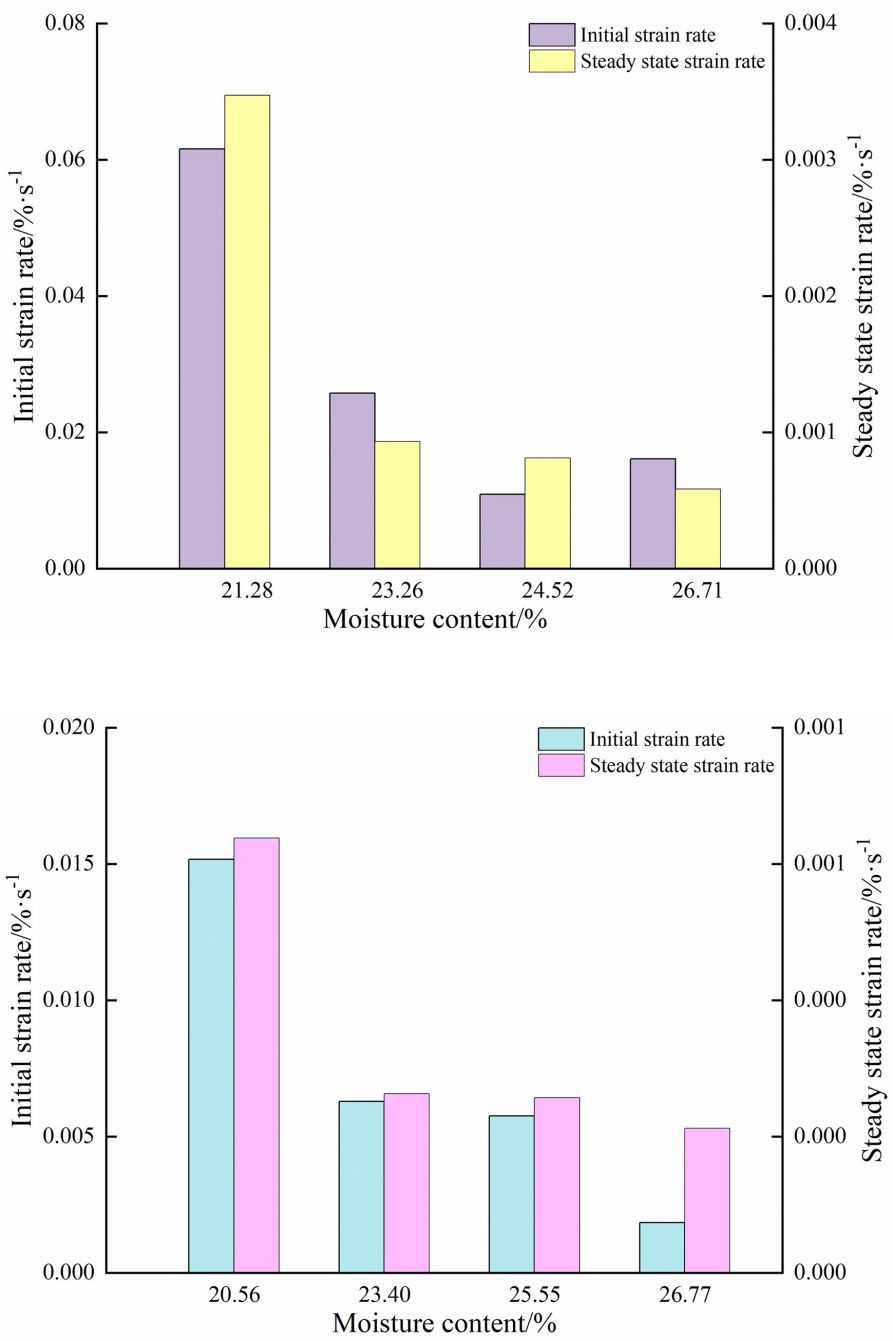
The creep rate of each treatment in the initial and steady-state stages. (a) No tillage; (b) Plough tillage.

From formula (7), it could be seen that the strain rate of paddy soil was composed of a viscoelastic strain rate and viscous strain rate. The change in curve of the strain rate with time during the compressive creep of NT and PT paddy soil was shown in Fig 8, which revealed that all samples had a distinct initial stage with an initially fast nonlinear strain rate that slowed over time. It could be deduced that the compression creep process of paddy soils of all moisture contents included a transient, decelerating, then steady creep stages. The initial value of creep rates *v*_Nm_ corresponding to NTT, NTS, NTN, and NTV were determined to be 0.062, 0.026, 0.011, and 0.016%·s^-1^ respectively, with steady-state creep stage rates *v*_Nn_ of 0.0035, 0.0009, 0.0008, and 0.0006%·s^-1^. The initial creep rates *v*_Pm_ corresponding to PTT, PTS, PTN, and PTV were 0.01517, 0.00629, 0.00576, and 0.00184%·s^-1^ respectively, with steady-state creep rates *v*_Pn_ of 0.00064, 0.000263, 0.000257, and 0.000212%·s^-1^. The corresponding creep rates of each treatment in the initial and steady-state stages were shown in Fig 9. As moisture content decreased, the strain rate exhibited a continuous decrease in the convergence and steady-state creep rate. Formula (9) was obtained by fitting the moisture content of paddy soil with the strain rate in the instantaneous creep stage and steady creep strain stage.


$$\left\{ \begin{array}{l} v_{\mathrm{Nm}}(w)=0.3083w^{2}-0.1565w+0.02\\ v_{\mathrm{Nn}}(w)=0.0172w^{2}-0.0088w+0.0011\\ v_{\mathrm{Pm}}(w)=0.02w^{2}-0.0114w+0.0016\\ v_{\mathrm{Pn}}(w)=0.0017w^{2}-0.0009w+0.0001 \end{array}\right.$$ (9)

Where: *v*_**Nm**_ is the initial creep rate (NT); *v*_**Nm**_ is the steady-state creep stage rate (NT); *v*_**Pm**_ is the initial creep rate (PT); *v*_**PN**_ is the steady-state creep stage rate (PT).

## Discussion

The purpose of this study is to study the creep properties of paddy soil by creep tests. The rheological parameters of paddy soil were obtained by creep model. The instantaneous elastic modulus, viscosity coefficient, delay elastic modulus, relaxation time, and delay viscosity coefficient of soil are important parameters of soil properties, including fluidity, deformation, failure, and adhesion. Changes in the tillage method and moisture content will affect these creep properties, altering the action mode and stress state of tillage tools and soil, thus influencing the tillage quality of soil, action nature of tillage tools, and growth of crop roots. The effects of the tillage method and moisture content on soil creep properties and soil tillage performance are discussed in the following sections.

### Effect of tillage method on creep properties of paddy soil

Different tillage methods affect the physical composition and soil structure of the surface layer of paddy soil [42], subsequently altering the soil rheology. Studies have shown that compared with traditional tillage, NT can provide better field water capacity and density for crops, especially in the topsoil [43]. In the present work, under the same sunning days, the moisture content of NT paddy soil decreased more slowly than that of PT. After the two fields were dried for 12 days, the moisture content of NTV was measured to be 21.28%, which is greater than 20.56% of PTV. The rheological parameters of the two paddy soils under the same sunning days were further compared and analyzed. Considering that the instantaneous elasticity modulus reflects the resistance of paddy soil to instantaneous deformation, the smaller elasticity modulus of NT paddy soil than that of PT paddy soil indicates its stronger resistance to deformation. The reason may be that the surface of NT land is covered by straw for years, which means that the surface is softer than that of bare-turned-arable land. Results of the delay elastic modulus showed that the hardness of NT paddy soil was less than PT paddy soil. Some studies have suggested that by returning a large amount of straw or mulch to the fields in conservation tillage, abundant food sources can be provided for soil animals, such as earthworms, that can subsequently improve soil hardness [44]. In the middle and lower reaches of the Yangtze River, under the conditions of mechanical disturbance and natural factors, the leaching effect of PT paddy soil causes the leakage and deposition of small particle size materials, such as upper surface clayey particles and fine silt particles, resulting in relatively compact and viscous paddy soil. Therefore, compared with NT paddy soil under the same load and environmental conditions, the viscosity of PT paddy soil is stronger and possesses a worse ability to return to its original size. Comparatively, the mean relaxation time of NT paddy soil showed to be greater than that of PT paddy soil, and the organic matter content of NT paddy soil increased due to successive straw mulching decay. Because organic matter is soft, porous and weak in plasticity, its strong water absorption capacity leads to decreased soil cohesion and increased soil elasticity [45]. As such, the increased organic matter in NT soil can improve the soil’s structure and physical properties, promote soil tilth improvement, and extend the tillage period [46]. The elastic hysteresis time of NT paddy soil was prolonged; thus, the suitable tillage period of paddy soil could be prolonged by increasing soil organic matter. Relevant studies have shown that straw is rich in carbon content, and the decomposition of straw after returned to the field will form pores, thereby increasing soil porosity and activated carbon content [25].

### Effect of moisture content on creep properties of paddy soil

The instantaneous elastic modulus, delay elastic modulus, and viscosity coefficient of paddy soil under each moisture content are inversely proportional to moisture content. The degree of soil deformation is related to the soil structure, pores between soils, and flow of water in the pores, which affect the composition and microstructure of soil [47]. When the soil moisture content is low and does not reach a saturated state, the proportion of air in soil pores increases, the soil surface becomes soft, and pore compression occurs relatively easily [48]. Therefore, the instantaneous elastic modulus of paddy soil shows a decreasing trend with increased moisture content. The delay elastic modulus determines the slow degree in the elasticity change of paddy soil in the stable creep process. Herein, during the stable creep of paddy soil under compression, the elastic deformation of NT paddy soil recovered faster than that of PT paddy soil, which may be due to the higher moisture content of NT paddy soil under the same drying treatment. With the increase of moisture content over time, water diffuses more thoroughly throughout the soil, which further reduces the soil strength by creating new pores and, thus, increases the soil elastic deformation [49]. Therefore, the moisture content is negatively correlated with the delay modulus of paddy soil. The moisture content in the optimal tillage period is generally between 20% and 30%. As moisture content increases, the distance between soil particles gradually increases. Due to the enhanced diffusion and inverted lubrication of water, the relative sliding friction between particles decreases and mobility increases, thus decreasing the viscosity coefficient of paddy soil [50].

Based on the above findings, it can be concluded that the relationship between moisture content and creep properties of paddy soil is complex, especially for the relaxation time and delayed viscosity coefficient of NT paddy soil. In the 5 fitting models, the fitting determination coefficients were all less than 0.90. Therefore, it is necessary to further study the influence of mechanical parameters under the combined action of viscoelasticity and the rheological coupling mechanism among soil particles, organic matter, and water in paddy soil to advance the research on the regulation of soil tillage.

## Conclusions

Based on Burgers model, this study analyzed the significant and quantitative relationship between the rheological parameters and moisture content of paddy soil under different tillage methods in the middle and lower reaches of the Yangtze River. Correlation analysis of the above mechanical properties revealed the different viscoelasticity of the internal structure of paddy soil with different tillage methods and moisture content. Under the three-year plough tillage and no tillage methods, the total creep deformation of paddy soil with different moisture content is based on elastic strain, viscous strain, and viscoelastic strain, while the strain rate is determined by the viscoelastic strain rate and viscous strain rate. The initial strain rate and steady strain rate of PT paddy soil were found to be lower than those of NT paddy soil.

This study provides an important theoretical basis for the dynamic analysis of soil in relation to the tillage machinery, soil compaction, farmland water, and soil regulation. Since soil is a kind of complex nonlinear viscoelastic plastic material, there is no mature rheological theory to explain its complex creep properties, which are influenced by numerous factors. The purpose of this study is to study the creep properties of paddy soil by creep tests. In addition to the moisture content and tillage methods mentioned in this paper, soil temperature, particle size distribution, crop root system, and trace element content are other pertinent factors of soil. Therefore, the creep properties of soil need to be further studied in combination with multidisciplinary knowledge to better understand and monitor soil for agricultural means.

## Supporting information

S1 Fig Soil sampling site (plough field). (TIF)

S2 Fig Soil sampling site (no tillage field). (TIF)
