# Supplementary figures and images for: Effects of moisture content and tillage methods on creep properties of paddy soil
Source: PLoS One. 2021 Jun 24;16(6):e0253623. doi: 10.1371/journal.pone.0253623 (PMC8224953; doi:10.1371/journal.pone.0253623)

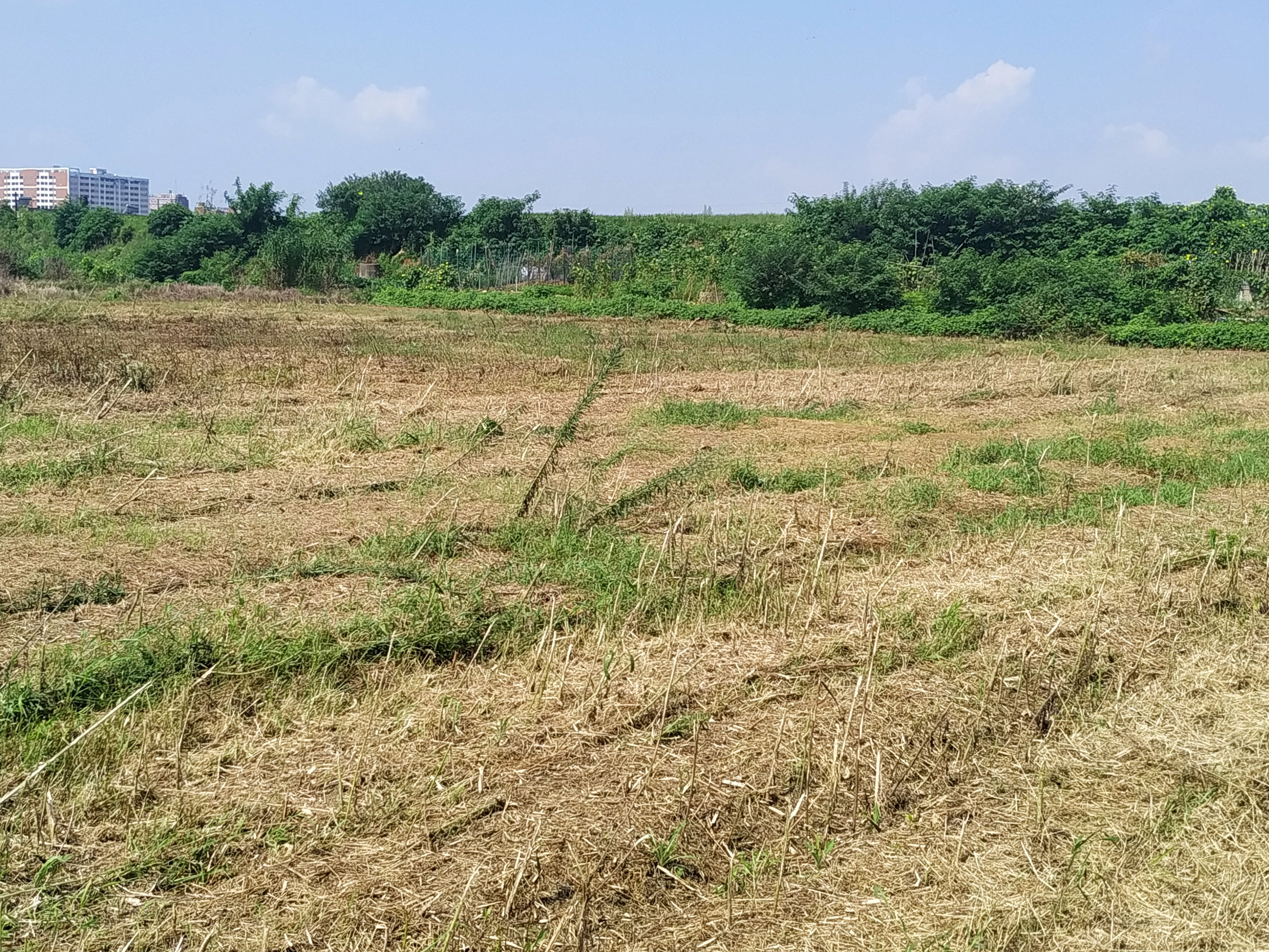

Supplement: S1 Fig — (TIF) [file pone.0253623.s001.tif]

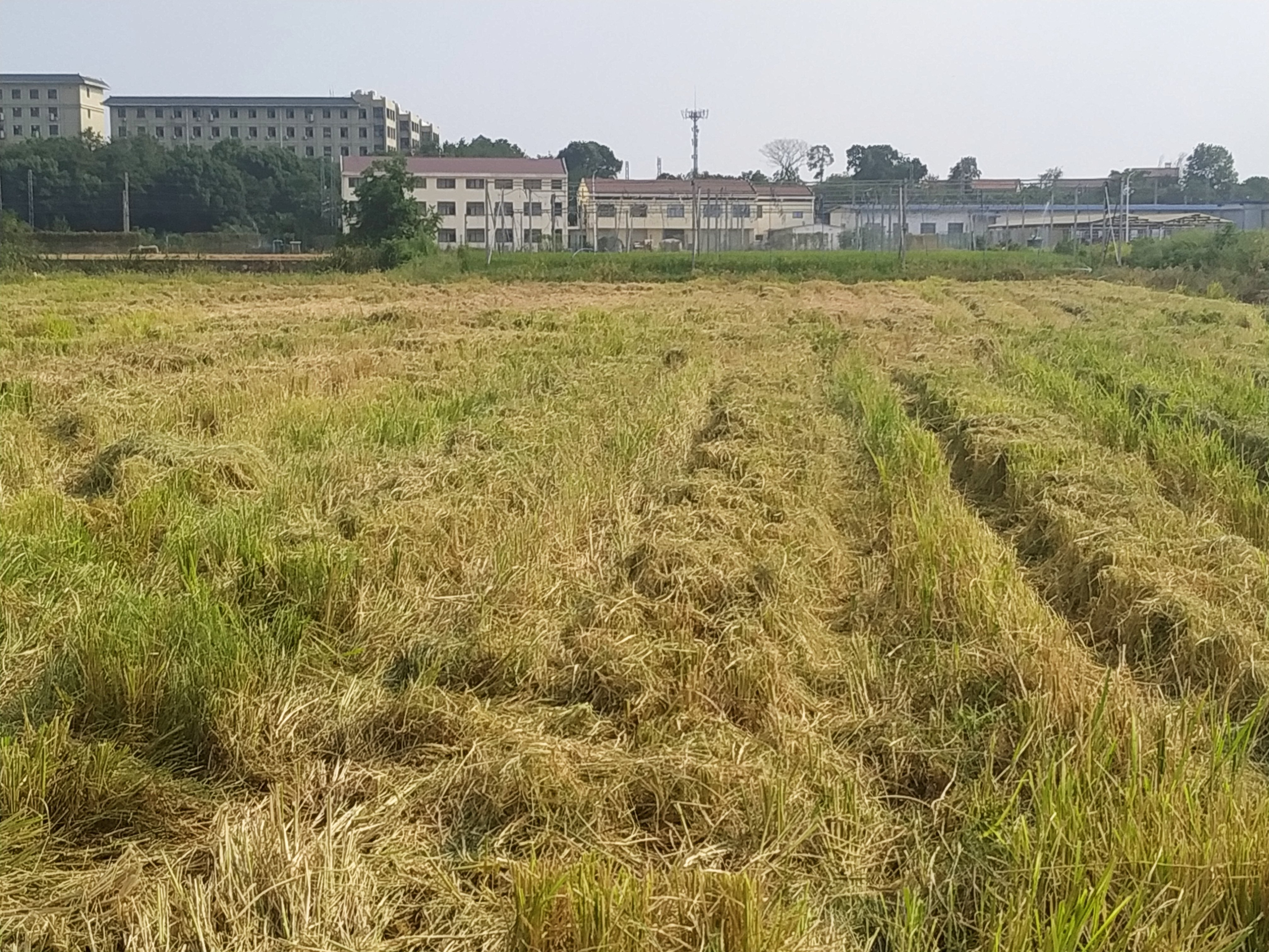

Supplement: S2 Fig — (TIF) [file pone.0253623.s002.tif]
